# Supplementary figures and images for: Glucose dysregulation in hospitalized non-critically ill patients with a suspected infection: A prospective study using continuous glucose monitoring
Source: PLoS One. 2026 Mar 2;21(3):e0343703. doi: 10.1371/journal.pone.0343703 (PMC12952634; doi:10.1371/journal.pone.0343703)

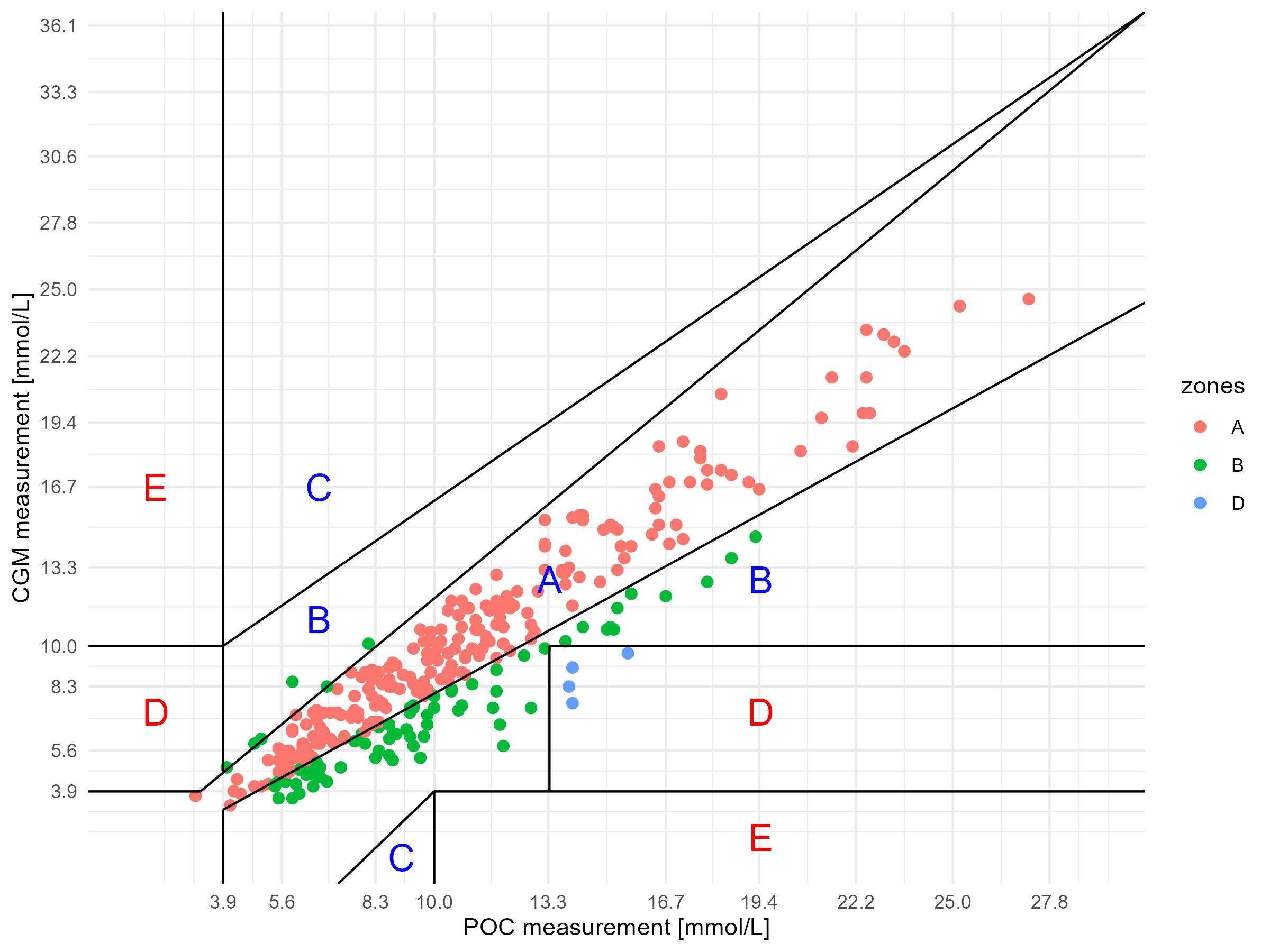

Supplement: S1 Fig — Distribution of matched glucose pairs across Clarke Error Grid zones (A–E), comparing point-of-care glucose values with FreeStyle Libre measurements. The analysis shows 75% of values in Zone A (clinically accurate), 24% in Zone B (benign errors), 0% in Zone C (errors potentially leading to unnecessary treatment), and 1% in Zone D (errors indicating failure to detect hypo- or hyperglycaemia). (TIFF) [file pone.0343703.s001.tiff]
