# Supplementary material for: Glucose dysregulation in hospitalized non-critically ill patients with a suspected infection: A prospective study using continuous glucose monitoring
Source: PLoS One. 2026 Mar 2;21(3):e0343703. doi: 10.1371/journal.pone.0343703 (PMC12952634; doi:10.1371/journal.pone.0343703)
Supplement: S1 Table — Summary of matched pairs, MARD values, and agreement percentages across different time intervals (0–24h, 24–48h, etc.) and glucose ranges (hypoglycemia, time in range, hyperglycaemia). (DOCX) [file pone.0343703.s002.docx]

# S1 Table. Detailed accuracy metrics of CGM vs. POC.

In this post-hoc analysis, we evaluated accuracy using several metrics: Mean Absolute Relative Difference (MARD), agreement rates within ±15%/15 mg/dL, ±20%/20 mg/dL, and ±30%/30 mg/dL.

|  | **Number of matched pairs** | **MARD (%)** | **%15/15** | **%20/20** | **%30/30** |
| --- | --- | --- | --- | --- | --- |
| **Overall** |  |  |  |  |  |
| CGM vs POC values | 312 | 14.1 | 60.9 | 75.6 | 91.0 |
| **Days** |  |  |  |  |  |
| 0 – 24h | 95 | 13.3 | 66.3 | 81.1 | 89.5 |
| 24 – 48h | 82 | 14.3 | 61.0 | 72.0 | 91.5 |
| 48 – 72h | 51 | 13.7 | 64.7 | 80.4 | 92.2 |
| 72- 96h | 48 | 15.4 | 45.8 | 75 | 89.6 |
| 96 – 120 h | 36 | 14.4 | 61.1 | 63.9 | 94.4 |
| **Glucose ranges** |  |  |  |  |  |
| Hypoglycaemia (<3.9 mmol/L) | 1 | 19.4 | 100 | 100 | 100 |
| Time in tight range (3.9-7.8 mmol/L) | 96 | 15.1 | 54.2 | 76.0 | 91.7 |
| Time in range (3.9 – 10.0 mmol/L) | 165 | 15.6 | 52.7 | 70.9 | 89.7 |
| Hyperglycaemia (>10.0 mmol/L) | 83 | 12.6 | 66.3 | 81.9 | 90.4 |
| Hyperglycaemia (>13.9 mmol/L) | 63 | 11.9 | 74.6 | 79.4 | 95.2 |
