# Supplementary material for: Glucose dysregulation in hospitalized non-critically ill patients with a suspected infection: A prospective study using continuous glucose monitoring
Source: PLoS One. 2026 Mar 2;21(3):e0343703. doi: 10.1371/journal.pone.0343703 (PMC12952634; doi:10.1371/journal.pone.0343703)
Supplement: Attachment — Submitted filename: Response to Reviewers.docx [file pone.0343703.s004.docx]

**Response to Reviewers**

**Title:** Glucose dysregulation in hospitalized non-critically ill patients with a ~~severe~~ suspected infection: a prospective study using continuous glucose monitoring

**Authors:** Anna D. Schoonhoven, Julia J. Bakker, Alessandra D. Di Mauro, Amarens van der Vaart, Riemer Been, A.P. van Beek, P. Choudhary, Hjalmar R. Bouma, Peter R. van Dijk

**Manuscript ID:** PONE-D-25-49860

**Corresponding Author:**

J.J. Bakker

Isala Zwolle

Email: [j.j.bakker@isala.nl](mailto:j.j.bakker@isala.nl)

**REVIEWER #1:**

**This manuscript by Schoonhoven et al., titled “Glucose dysregulation in hospitalized non-critically ill patients with a severe infection: a prospective study using continuous glucose monitoring” investigated the prevalence and course of dysglycaemia in non-critically ill adults hospitalized for severe infection using CGM, the authors find that in 90 patients, dysglycaemia is common in both diabetic and non-diabetic inpatients with infection and CGM could improve detection and management. The study provides interesting pilot data but the small sample size and low number of clinical events limit the robustness of conclusions, particularly regarding associations between dysglycaemia and outcomes. Larger multicenter studies are needed to confirm these findings. Additionally, several methodological and interpretative issues need to be addressed.**

We thank reviewer #1 for the constructive feedback. We agree that the small sample size and low number of clinical events limit the generalizability of our findings regarding clinical outcomes, and we appreciate the opportunity to clarify methodological and interpretative aspects. In the points below, a more detailed response to this issue is provided; however, we would like to indicate that we acknowledge the limited sample size, recognize that this indeed affects the interpretation of our findings, and have therefore moderated the tone in the discussion and conclusions. In line with this acknowledgment, we have revised the Conclusions to avoid overinterpretation of the findings by removing *“CGM offers a viable option for this as it could improve the detection and management of dysglycaemia and lead to better outcomes”* and replacing it with *“CGM may offer a viable tool for improving the detection of dysglycaemia and could serve as a basis for future interventional studies aimed at optimizing in-hospital glucose management.”* (Revised Manuscript with Track Changes, line 372–374).

Limitations related to sample size and statistical power are now explicitly addressed in the Discussion (Revised Manuscript with Track Changes, lines 290–294 and 359–362).

**In the method section of study design, Inclusion and exclusion criteria are clear, but justification for excluding corticosteroid users should be elaborated, does this exclusion bias the sample toward less severe infections?**

Corticosteroid therapy was excluded because of its substantial and variable effects on glucose metabolism, which could confound the primary outcome (occurrence of dysglycaemia). Importantly, excluding corticosteroids does not necessarily bias the study population toward patients with less severe infections. Corticosteroid use is not limited to severe infections; many people receive them for chronic conditions such as autoimmune diseases, asthma, or COPD. As a result, steroid users reflect a wide range of illness severity rather than predominantly severe infectious presentations. In addition, at our center a substantial proportion of corticosteroid users are organ transplant recipients who are instructed to seek early medical evaluation and are often admitted before severe systemic illness develops. Therefore, the exclusion of corticosteroid users is unlikely to systematically remove patients with more severe infections.

This is elaborated and clarified in the Materials and Methods section (Revised Manuscript with Track Changes, line 127-128): *“In addition, we excluded persons who used glucocorticosteroids, as these drugs have substantial and variable effects on glucose metabolism that could confound primary outcome.”*

**Please clarify whether “severe infection” per inclusion criteria.**

We revised our terminology from ‘severe infection’ to ‘suspected infection’, as this more accurately reflects our inclusion criteria based on at least one of the predefined clinical indicators. This approach reflects real-life emergency department practice, where treatment and triage decisions are based on clinical suspicion.

This is stated in the Materials and Methods section (Revised Manuscript with Track Changes, lines 110–112): *“For the present study, we included all adult patients (≥18 years) admitted through the emergency department with a clinical suspicion of infection, as determined by routine ED practice.”*

**In Statistical Analysis, it is stated that p<0.05 was considered significant, but multiple comparisons were made without correction. Please comment on whether a correction (e.g., Bonferroni or FDR) was considered. Was any regression modeling performed to adjust for potential confounders such as age, BMI, or diabetes status when exploring associations with dysglycaemia?**

We agree that multiple comparisons require careful consideration. We did not apply formal correction for multiple comparisons because the baseline variables in Table 1 are descriptive and not intended for hypothesis testing. For the glucose-derived metrics in Table 3, multiplicity adjustment (e.g., Bonferroni or FDR) was considered, but these metrics are physiologically correlated rather than independent outcomes, and strict correction methods would therefore be overly conservative [32].

In addition, multivariable regression to adjust for potential confounders such as age, BMI or diabetes status was considered not feasible due to the small number of adverse clinical events (n=9). This would not allow for adequately powered adjusted models [33]. Therefore, results are presented as univariate exploratory associations.

This has been clarified in the Statistical Analysis section (Revised Manuscript with Track Changes, line 180-184): *“Given the exploratory nature of this study and the limited sample size, no formal adjustment for multiple comparisons was applied [32]. Regression analyses to adjust for confounders such as age or BMI were considered but not performed due to the small number of adverse clinical events [33]. Instead, exploratory univariate regression analyses were conducted to assess associations between dysglycaemia and clinical outcomes.”*

This limitation is also acknowledged in the Discussion (Revised Manuscript with Track Changes, lines 270–271 and 352–353).

**In the discussion, the authors conclude that CGM may “allow earlier intervention” but as the study was blinded and observational, this inference is speculative. Please temper this statement or support it with cited intervention studies.**

Regarding the statement that CGM may “allow earlier intervention” in the Discussion, we agree that our blinded observational design does not permit causal inference. We have therefore tempered this wording to reflect that CGM may enable earlier detection of dysglycaemia, which could inform future interventional research rather than directly allowing earlier intervention. This change has been implemented consistently throughout the manuscript.

This is most clearly reflected in the revised Conclusions (Revised Manuscript with Track Changes, lines 372–374): *“CGM may offer a viable tool for improving the detection of dysglycaemia and could serve as a basis for future interventional studies aimed at optimizing in-hospital glucose management.”*

**The reported 55% rate of hypoglycaemia (including in non-diabetic patients) seems high. Could sensor compression or first-day calibration bias explain part of this? Consider adding a sensitivity analysis excluding the first 24 hours of CGM data.**

First-day sensor behavior, particularly the known under-reading of interstitial glucose near the hypoglycaemia threshold in the FreeStyle Libre Pro iQ, may contribute to an overestimation of level-1 hypoglycaemia. To address this concern, we conducted an additional analysis examining the distribution of hypoglycaemic events across sensor days. The absolute number of hypoglycaemic readings was highest on day 1 (98 events), but because the number of patients with available data declines over time, we normalized hypoglycaemia counts to the number of patients contributing data per day. The resulting rates were relatively stable across days (day 1-5: 2.8, 2.33, 2.50, 1.81 and 1.85 events per day, respectively), indicating that although day 1 shows slightly more events, hypoglycaemia was not disproportionately concentrated in the first 24 hours. This pattern is visualized in the figure below, where the first subplot shows the number of hypoglycaemic events and the number of patients per day, and the second subplot shows the corresponding normalized hypoglycaemic rates. Together, these findings suggests that excluding the first day would be unlikely to alter the overall conclusions and would omit clinically relevant data on early infection-related glucose variability. We have now incorporated these findings and expanded the Discussion accordingly, clarifying that most hypoglycaemic episodes were brief, mild, and likely reflect physiological or sensor-related variation rather clinically significant hypoglycaemia (Revised Manuscript with Track Changes, line 322-330).

Figure 1: The upper panel shows the absolute number of hypoglycaemic events per day alongside the number of participants contributing data. The lower panel displays the normalized number of hypoglycaemic events per patient per day.

**REVIEWER #2:**

**The topic is clinically relevant and timely, as glucose instability in acute infection is common but under-characterized outside the ICU setting. The prospective design and adherence to STROBE standards are strengths. However, the study as presented remains primarily descriptive and exploratory. The analyses are insufficiently detailed; the comparisons lack statistical rigor, and several methodological and interpretive limitations reduce the strength of the conclusions. Substantial revision is required before this manuscript can be considered for publication.**

We thank reviewer #2 for the critical and constructive comments. We have addressed each point as thoroughly as possible within the constraints of the available data. Given the observational and exploratory nature of this study, we have aimed to appropriately temper our analyses and conclusions and to explicitly acknowledge the study’s limitations.

**1. The study relies mainly on descriptive and univariate comparisons between patients with and without diabetes. To enhance interpretability, apply multivariate regression (logistic or linear, as appropriate) to evaluate associations between dysglycaemia metrics (such as mean glucose, time in range) and outcomes (ICU admission, mortality, hospital stay). Also consider adjusting for potential confounders such as age, BMI, CRP, and infection severity.**

We agree that multivariate regression could improve the interpretability of associations between dysglycaemia and clinical outcomes. However, in our dataset, the number of outcome events was limited: only five participants required ICU admission and six died within 30 days, resulting in a combined total of nine outcome events. Given the limited number of outcome events, multivariable regression models would be prone to overfitting and be statistically unreliable [33]. Therefore, we limited our analyses to exploratory univariate comparisons, which showed no significant differences between participants with and without dysglycaemia.

This rationale has been clarified in the Statistical Analysis section (Revised Manuscript with Track Changes, lines 180–184) and acknowledged as a limitation in the Discussion (lines 291–295).

**2. Report effect sizes and 95% confidence intervals, not only p-values.**

We have revised our analyses to present univariate regression analyses for the associations between dysglycaemia and clinical outcomes. Specifically, we now report effect sizes with 95% confidence intervals for both the continuous outcome (length of stay using linear regression) and binary outcome (composite 30-day mortality or ICU admission, using logistic regression).

This has been clarified in the Results (Revised Manuscript with Track Changes, line 260-265): *“Univariate linear regression showed that participants with dysglycaemia had a non-significant increase in length of hospital stay of 1.33 days compared with those without dysglycaemia (β = 1.33, 95% CI –1.20 to 3.85, p = 0.30). Univariate logistic regression indicated that dysglycaemia was not significantly associated with the composite outcome of 30-day mortality or ICU admission (OR = 0.42, 95% CI 0.10–2.16, p = 0.25).”*

**3. The study concludes that dysglycaemia was common but not associated with clinical outcomes. Given the small number of adverse events, this result may be due to limited power rather than a true absence of association. The authors should clearly state the low event rate as a limitation.**

We agree that the lack of observed associations between dysglycaemia and clinical outcomes may be related to limited power due to the small number of adverse events. This does not allow multivariable analyses to adjust for potential confounders without substantial risk of overfitting.

This point has been clarified as a limitation in the Discussion (Revised Manuscript with Track Changes, line 360-363): *“This led to a small number of adverse clinical events which led to limited statistical power to detect large effects. The absence of significant associations between dysglycemia and clinical outcomes should therefore be interpreted with caution [39].”*

**4. Discuss whether dysglycaemia might serve as an early biomarker for clinical deterioration, rather than an independent outcome predictor.**

We agree that dysglycaemia could serve as an early biomarker of clinical deterioration. However, given our limited event rate and the design of the study, which was not intended to investigate underlying physiological mechanisms, we cannot determine whether dysglycaemia reflects early metabolic stress or functions as an independent outcome predictor. Although we certainly acknowledge this point, we are - given the previously noted and discussed limitations of this observational, primarily descriptive study -somewhat cautious about making very strong statements in this regard. We have therefore revised the Discussion to frame dysglycaemia as a potential early marker of worsening clinical status, while emphasizing that this interpretation is hypothesis-generating and requires confirmation in larger, adequately powered cohorts (Revised Manuscript with Track Changes, line 291-295). To contextualize this concept, a brief explanation has also been added to the Introduction (Revised Manuscript with Track Changes, line 72–75).

**5. The analysis would be more informative if additional variables were included such as type and site of infection, nutritional intake, IV glucose, and insulin dosing during admission (noting that severity scores like SIRS/qSOFA are already reported at baseline). Without these, the ability to interpret the mechanisms or contributors to dysglycaemia is limited.**

We agree that these variables could provide valuable insights into the mechanisms or contributors underlying in-hospital dysglycaemia. Unfortunately, detailed data were not systematically or reliably recorded in our cohort (particularly nutritional intake and site or type of infection) and therefore could not be included. We have added this limitation to the Discussion and emphasized that future studies should incorporate these variables to better identify contributors to dysglycaemia (Revised Manuscript with Track Changes, line 354-357).

**6. Expand discussion on why hypoglycaemia and hyperglycaemia patterns differ between groups.**

Differences in glycaemic patterns between persons with and without diabetes likely arise from several physiological and treatment-related factors. In individuals with diabetes, the persistence of hyperglycaemia throughout admission is associated with underlying insulin resistance as described in the Introduction. Hypoglycaemia in this group is plausbily related to insulin therapy in the context of rapidly changing insulin requirements during infection. In persons without diabetes, the observed hyperglycaemia may reflect metabolic disruption during acute illness by an infection-related stress response, including counter-regulatory hormonal activation. Because this study was designed to characterize the occurrence and timing of dysglycaemia rather than to determine its causes, and detailed data on nutritional intake or medication adjustments were not available, these interpretations remain cautious. Nevertheless, these exploratory results provide a plausible physiological context that supports the relevance of CGM for detecting clinically meaningful glucose disturbances in non-critically ill patients with infection.

We have added this to the Discussion (Revised Manuscript with Track Changes, line 278-281): *“The observed differences in hypo- and hyperglycaemia patterns between patients with and without DM are likely related to physiological and treatment-related factors such as persistent insulin resistance in DM or stress-induced dysregulation in persons without DM.”*

**7. Provide a mechanistic rationale connecting infection-related stress responses with CGM-detected glycaemic patterns.**

We agree that providing a mechanistic rationale helps to strengthen the interpretation of CGM-detected glycemic patterns compared to infection-related stress responses in both groups and we have expanded the Introduction briefly (Revised Manuscript with Track Changes, line 72-75): *“Together, these processes reflect the acute metabolic stress and systemic inflammatory response induced by infection, which may provoke early disturbances in glucose homeostasis even in individuals without diabetes.”*

The described components of infection-related stress response offer an explanation for the glycemic variability captured by CGM in our cohort. However, this study was not designed to distinguish between these mechanisms. Detailed data on inflammatory markers, nutritional intake, medication dosing, and infection characteristics were not available, limiting mechanistic interpretation. We emphasize that these findings should be viewed as hypothesis-generating and that future cohorts are required. This is clarified in the Discussion (Revised Manuscript with Track Changes, line 291-295).

**8. Change the conclusion that CGM “may improve outcomes,” which is not supported by the presented data.**

Thank you for this point. We agree that our data do not support the claim of improving outcomes. The statement was tempered by revising the Conclusion. Causal language was removed (Revised Manuscript with Track Changes, lines 370–371) and CGM was reframed as a tool for improved detection of dysglycaemia and a basis for future interventional studies (Revised Manuscript with Track Changes, lines 372–374).

**9. Define “severe infection” operationally—were diagnoses microbiologically confirmed or purely clinical?**

Thank you for this comment. We revised our terminology in the whole article from ‘severe infection’ to 'suspected infection’ as this more accurately reflects our inclusion criteria based on at least one of the predefined indicators. Diagnoses were not microbiologically confirmed, because this is a retrospective variable not available at the time of inclusion at the emergency department. The approach of defining our study population by clinical suspicion criteria reflects real-life practice, where treatment and triage decisions are based on clinical suspicion without microbiological confirmation. More importantly, negative microbiological samples could be unreliable as the use of antibiotics before the sample was taken contaminates.

This is described and clarified in the Materials and Methods section (Revised Manuscript with Track Changes, line 109-121).

**10. Clarify whether HbA1c data were unavailable or simply not collected; this affects interpretation of undiagnosed diabetes.**

We appreciate this comment. HbA1c values were not collected in this study, as the primary aim was to assess in-hospital glycaemic variability using continuous glucose monitoring rather than to characterize pre-existing glycaemic status. We acknowledge that the absence of HbA1c data limits the ability to distinguish between newly developed dysglycaemia and pre-existing diabetes or impaired glucose control. This limitation has been acknowledged in the Discussion (Revised Manuscript with Track Changes, line 356-362) where we note that the lack of HbA1c measurements prevents assessment of long-term glycaemic control and may have led to an underestimation of previously undiagnosed diabetes among participants.
